# Coping Strategies with Genito-Pelvic Pain/Penetration Disorder: A Qualitative Study

**DOI:** 10.1155/2023/5791751

**Published:** 2023-12-16

**Authors:** Mojdeh Banaei, Vahid Mehrnoush, Nasibeh Roozbeh, Nourossadat Kariman

**Affiliations:** ^1^Mother and Child Welfare Research Center, Hormozgan University of Medical Sciences, Bandar Abbas, Iran; ^2^Midwifery and Reproductive Health Research Center, School of Nursing and Midwifery, Shahid Beheshti University of Medical Sciences, Tehran, Iran

## Abstract

**Background:**

Genital/pelvic pain penetration disorder (GPPPD) decreased mental and physical functioning, reduced quality of life, and reduced feelings of inadequacy and worthlessness, all of which impair the ability of women with GPPPD to enjoy sex. This qualitative study was conducted to identify which factors can reduce sexual stress and help Iranian women cope with GPPPD.

**Methods:**

This qualitative study was conducted through the participation of 18 women with GPPPD diagnosed by a sexologist and using DSM-IV diagnostic criteria from March to July 2022, Iran. The samples were selected using the purposive sampling method and considering the maximum variation. The semistructured question guide was used as a data collection tool and data collection continued until data saturation was reached. The collected data were analyzed using conventional content analysis approach.

**Results:**

Data analysis led to the emergence of three main themes: “problem-focused coping” which included the three categories of received social support, problem self-control, and penetration replacement; “emotion-focused coping” which included three categories: a couple's negative reaction to the problem, attachment disorder, and surrendering the problem; and “treatment-seeking” which consisted of searching and choosing a therapist to solve the problem, ineffective medical approaches, and ineffective nonmedical approaches.

**Conclusion:**

Coping strategies in women with GPPPD were classified as “problem-focused coping,” “emotion-focused coping,” and “treatment-seeking.” These findings indicate a need for GPPPD information and education, as well as a need for healthcare professionals to actively inquire about sexual problems and commit to serious treatment efforts. Cultural interventions that promote sexual pleasure can aid in the management of GPPPD.

## 1. Introduction

Sexual health is a fundamental aspect of personal health that affects all people of all ages and life stages [1]. The World Health Organization recently defined sexual health as “the experience of the ongoing process of physical, psychological, and sociocultural well-being related to sexuality” [2]. In fact, sexual health is not simply the absence of disease or dysfunction but an important and integral part of being human [3]. Currently, sexual pain disorders are called as genital/penetrative pain disorder in the DSM-5 [4]. Genital/pelvic pain/penetration disorder (GPPPD) is a sexual problem defined as persistent or recurrent problems accompanied by one or more of the following symptoms: inability to penetrate the vagina during intercourse, significant pelvic or vulvovaginal pain during intercourse or attempting intercourse, and anxiety or fear during vaginal penetration [5]. GPPPD is diagnosed when one of the above criteria is met for at least six months and clinically significant symptoms are present [6]. An overlapping disorder not based on DSM classification is vulvodynia, or chronic unexplained vulvar pain, which is also associated with pelvic floor muscle dysfunction and superficial dyspareunia. These common gynecological pain conditions cause serious sexual, psychological, and relational disturbances that cause as much pain to the patient and their partner as the pain itself [4].

Sexual pain is a common problem [5]. According to the results of 29 studies with women aged 40–80 years, the prevalence of sexual pain in women in the Middle East, East Asia, and Southeast Asia is nearly 21%, 31.6%, and 29.2%, respectively [7]. In a study by Alizadeh et al. in Iran, the prevalence of GPPPD was estimated to be 16% [8]. In primary health care in Spain, one in nine women suffer from these disorders; the percentage of women with GPPPD in this study was 11.23% (vaginismus 5%, shooting pain 8.33%, and dyspareunia 16.45%) [9]. GPPPD has a multifactorial and complex etiology, with biological, psychological, and relational factors interacting to maintain and sustain a woman's pain response. In the absence of acute injury, what may initially be an adaptive nociceptive response to peripheral tissue injury may gradually transform into neuropathic and/or inflammatory pain. This maladaptive pain impairs sexual function, particularly as the central nervous system becomes more involved in pain sensitization [6, 10]. As a result, GPPPD should be evaluated from a biopsychosocial standpoint rather than as a purely psychogenic problem [11].

Sexual pain impairs overall well-being as well as depression-related mental health symptoms [12]. For example, Schneider et al. found dyspareunia in adolescent girls and young adults, decreased mental and physical functioning, reduced quality of life, and reduced feelings of inadequacy and worthlessness, all of which impair their ability to enjoy sex [13]. Almost half of women with chronic pelvic pain also suffer from other painful conditions such as fibromyalgia, interstitial cystitis, or irritable bowel syndrome [14]. It is also linked to other sexual dysfunctions such as female sexual interest/arousal disorder and decreased sexual satisfaction [15].

The impact of sexual pain on women's psychological well-being, sexual well-being, and sexual functioning requires the identification and application of effective coping strategies to experience pleasurable sexual relationships [16]. In terms of coping, Herzberg proposed that the term “coping mechanism” refers to a variety of ways in which individuals (along with their partner and those around them) may interact when confronted with stressors. Individuals typically respond by concentrating on their emotions and the problems they are attempting to solve [17]. In a study among Swedish women, nearly half of those who experienced pain during vaginal sex reported continuing to have sex despite the pain. This can be interpreted as endurance-related coping [18]. Another study showed that fear responses (vaginal tension) and fear-avoidance beliefs about sexual activity were strongly associated with pain, suggesting that fear-related avoidance may be another coping strategy [19].

A study of women with endometriosis found that they used both adaptive and nonadaptive coping strategies and that suppressed own emotions, catastrophic pain, and passive coping styles were associated with pain; emotional and avoidant coping styles were associated with poorer mental health, while positive or emotion-focused coping styles, distanced, and rational coping styles were associated with better mental health [20]. Malone et al. found that Black women use a variety of multiple proactive coping strategies for sexual pain relief, including nonpenetrative activities, foreplay, arousal-enhancing tools, physical adjustments, sexual intimacy, and communication [16]. However, little research has been done on the coping strategies used by women with GPPPD to cope with sexual health problems. In other words, the purpose of the study is to identify which factors can reduce sexual stress and help people cope with GPPPD. These findings can help therapists and patients to design more effective pain relief interventions. Therefore, in this study, we look at how Iranian women with GPPPD cope and overcome sexual pain.

## 2. Materials and Methods

### 2.1. Research Design

Based on the criteria proposed by Graneheim and Lundman, a qualitative study was conducted using the conventional approach to content analysis. We used the Consolidated Criteria for Reporting Qualitative Research (COREQ). The checklist consists of 32 items divided into three categories: (i) group research and reflection, (ii) study design, and (iii) data analysis and reporting [21].

### 2.2. Study Subjects

This study was conducted from March to July 2022 to determine the need of 18 women with GPPPD in Iran. The research setting included sexual health clinics in Bandar Abbas. The following criteria were used to determine inclusion: Iranian married women aged 18–50 with GPPPD diagnosed by a sexologist and using DSM-5 diagnostic criteria, with no other physical or acute or chronic mental disorders (based on self-report). Due to ethical issues and data availability, just married women were included in this study.

GPPPD was diagnosed using the following DSM-5 criteria:

- Persistent or recurring problems with one or more of the following for at least 6 months
- Inability to penetrate the vagina or intimacy
- Severe vulvar, vaginal, or pelvic pain during vaginal penetration or intercourse
- Fear or anxiety about vulvovaginal pain, pelvic pain, or vaginal penetration
- Marked contraction of the pelvic floor during vaginal penetration
- Caused significant clinical distress to the individual.

The disorder is not associated with any significant, distressing, or severe nonsexual psychological problem or relationship disorder nor is it associated with any drugs or other medical conditions. The only exclusion criterion was the participant's willingness to withdraw from the study.

### 2.3. Interview Outline

The semistructured question guide was used as a data collection tool. By the face-to-face method, the interview began with open-ended questions about women with GPPPD's coping strategies, such as “What is your sexual disorder?” Explain. “What did your partner do to solve this problem?” “What strategies have helped you to adapt to this disorder? and” “What strategies do you suggest to improve this disorder?” During the interview, whenever the researcher felt the need to delve deeper and obtain more explanations, she asked probing questions such as “How?” “What do you mean?” and “Please explain” more if you can. Each interview lasted 45–60 minutes and all interviews were recorded (Table 1). Notes and memos were also used to collect data. In total, we invited 20 participations, and 2 women with GPPPD declined to participate in the interview. Although data saturation was reached in 15 interviews, three more interviews were conducted to increase certainty.

### 2.4. Data Collection

For data collection and interviews, the researcher referred to preferred sexual health clinics in Bandar Abbas, Iran. A targeted selection interview was conducted, with maximum diversity in terms of age, level of education, employment status, and length of marriage considered. Women with GPPPD were invited to participate in the study using the direct method. Before conducting the interviews, the researcher explained to the participants the purpose of the study, confidentiality of the information, and voluntary participation and obtained written and verbal informed consent for study participation and voice recording.

All interviews were conducted by the first author; she holds a Ph.D. in Reproductive Health and has passed qualitative research courses. The data collection and analysis were supervised by a professor who specialized in qualitative research and also lectures at medical universities. An in-depth, semistructured individual interview with open-ended questions in Persian was used as the data collection method. All interviews were conducted in private settings and also in places such as the workplace, which the participants preferred, and the participants were given nicknames. If a participant refused to allow voice recording, notes were taken instead.

### 2.5. Rigor

The rigor and reliability of the data were assessed using the Guba and Lincoln criteria. The credibility of the study was validated by allowing adequate time for data collection, researcher triangulation, expert evaluation, long-term involvement of researchers in the data, and participant selection (member review). Therefore, both participants were given interview texts as well as extracted codes to ensure that the results are consistent with their experiences. To increase reliability, the research team commissioned an external auditor to audit all phases, such as data collection and analysis. The external reviewers were research independent and specialists in qualitative research.

In addition, other study members reviewed the interview text and coded interviews, and any disagreements were addressed and resolved through discussion among study members. Various sources (women with GPPPD, spouses, and specialists) were used for the triangulation. In addition to interviews, field notes and memos were also used for data collection. The samples were selected with the greatest amount of diversity for transferability and comprehensiveness. To ensure data verifiability, the researcher discarded all assumptions and considerations and thoroughly documented all phases of the research, including data collection, data analysis, and code development.

### 2.6. Data Analysis

Data were analyzed in MAXQUDA10 concurrently with data collection using a conventional content analysis approach based on the criteria proposed by Graneheim and Lundman. After transcribing the recorded interviews, they were carefully reviewed by the first author (M.B), a Ph.D. of reproductive health who had completed qualitative research courses, to ensure an accurate understanding of the interview contents. The whole process of data analysis was supervised by N.K., an expert in qualitative research and faculty members of Shahid Beheshti University of Medical Sciences.

In addition, other research members reviewed the coded interviews, and any disagreements were discussed and resolved. This method encourages reflection by examining an individual's own construction of knowledge, effectively improving overall transparency and trustworthiness while also ensuring the methodological soundness and practical relevance of the research [22]. All stages of the research were recorded very precisely for the possibility of an audit. All elements of trustworthiness, including credibility, dependability, and transferability, were considered in this study. The researcher abandoned all of her assumptions and the results were presented using thoughts and quotations from the participants.

After reviewing the text of the interview, the text was divided into meaning units. Meaning units were condensed while preserving the meaning and were labeled with codes. Similar codes were then categorized into subcategories, and the subcategories were further classified into a category based on common properties. The latent content of the related categories was eventually developed into a theme. In this process, we used an external auditor to allow for investigating the process of data analysis. In addition, if there was any disagreement, the research team discussed and resolved it.

### 2.7. Ethical Consideration

This study was approved by the Ethics Committee of Hormozgan University of Medical Sciences (IR.HUMS.REC.1402.039). Confidentiality of the data collected was approved and written informed consent was obtained from all study participants. In addition, participants were informed that they are free to refuse to participate or withdraw at any stage of the research process. All interviews were recorded with the consent of the participants and all audio files were securely stored on password-protected computers.

## 3. Results

**Figure 1:**
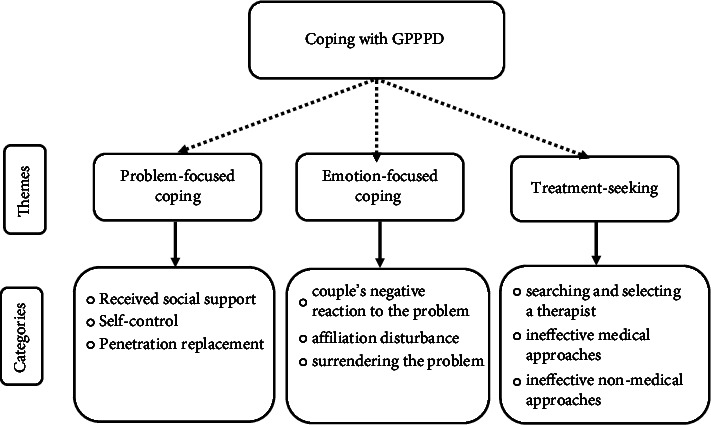
Theme and categories.

In total, 18 women with GPPPD participated in an interview. The mean age of women was 32.77 (range 19–49 years) and the mean relationship duration was 5.61 years (range 2–18 years). 50% of the women were housewives (*n* = 9) and 50% were employees (*n* = 9). 72.22% of women had a university education (*n* = 13). Data analysis revealed three major themes: “problem-focused coping,” “emotion-focused coping,” and “treatment-seeking.” Figure 1 depicts the themes and categories.

### 3.1. Problem-Focused Coping

Problem-focused coping keeps the person's attention focused on the problem and leads to solving the problem; as a result, they can ultimately produce positive emotion. This theme consisted of three categories of received social support, problem self-control, and penetration replacement.

#### 3.1.1. Received Social Support

Participants in this category mentioned the spouse's emotional support, respect for the spouse in sexual relations, and the support of the surrounding people. Receiving social support from others, particularly the spouse, increases the number of people coping with sexual problems.

Some participants in the interview mentioned the emotional support of the spouse.

*“My husband helps me greatly in this matter; he talks to me before the intimacy and comforts me, hugs me constantly, and expresses his love for me so that I feel he is always on my side and helps me in resolving this problem.” (23-year-old woman, bachelor's degree, 4 years married)*

The majority of participants stated that their spouse respected their feelings in sexual relationships.

*“My husband claims that I don't want to continue the intimacy because I saw the fear and pain in your eyes. “You can't do this by force,” he says.” (19 years old woman, 2 years of marriage)*

Some participants mentioned the support of those around them.

*“My mother and sister really helped me compassionately, they didn't even say a sad word to me during this time, and they just gave me self-confidence and said we know you can and your problem will be solved, I was very calm with their words.” (40-year-old woman with a master's degree who has been married for 12 years)*

#### 3.1.2. Problem Self-Control

Participants in this category stated that to control their problem, they had done things like doing muscle relaxation exercises before penetration, avoid negative thoughts, try to increase sexual awareness, and escape from the problem. Some participants mentioned muscle relaxation before penetration.

*“When I want to do penetration because I see my leg or vaginal muscles tighten, I take a deep breath before that to clear my mind and relax and tighten my muscles every 2-3 seconds, however, it does not work.” (24-year-old woman with a post-graduate diploma who has been married for three years)*

Another issue raised by many participants in this subcategory was the need to avoid negative thoughts.

*“I was always trying to calm my mind, I told myself that women can give birth to a child, this is a simple thing compared to that, so I can definitely do it, but again, even though I was talking to myself and convincing myself,” I still don't know what exactly I'm afraid of, and what that fear is (32-year-old woman, bachelor's degree, 4 years married)*

The majority of participants mentioned efforts to raise sexual awareness.

*“I was searching about the type of hymen and if there is any solution, when I came across the word dyspareunia, and I saw that it is very similar to my situation.” (30-year-old woman with a master's degree who has been married for 5 years)*

Several contributors mentioned being able to avoid the problem in this subcategory.

*“I suffered for 6 years. To escape this pain, I immersed myself in studies and work; I also earned a bachelor's degree from a prestigious public university, and I was the top student in my class. I mean, I got involved in studies and work to distract myself from my problem.” (34-year-old wo woman man with a bachelor's degree who has been married for two years)*

#### 3.1.3. Penetration Replacement

Replacing penetration was one of the participants' experiences in dealing with this sexual disorder. Some participants stated that they stopped having sex with their partner or performed intercrural sex, oral sex, and anal sex to avoid the pain or fear they feel after vaginal intimacy.

Many participants stated in the interview that they had stopped having sex.

*“When my husband gets closer to me, I feel intense fear and pain; I don't even want him to hug, cuddle, or kiss me… I assumed that every hug would result in intercourse, which is why we were uninterested in sexual intimacy.” (26-year-old woman, bachelor's degree, 3 years married)*

Some people in this subcategory mentioned intercrural sex.

*“We wanted to have sex, and we did, but not through penetration, because I was in a lot of pain and my husband could not see that I was hurting, and our relationship suffered as a result. Our intimacy was limited to cuddling and thigh sex.” (36-year-old woman, high school, 4 years married)*

Oral sex was mentioned by several participants.

*“Instead of penetration, we usually have oral sex, that way we satisfy ourselves.” “Oral sex is very enjoyable for both of us because his tongue does not go inside my vagina, so he has pleasure, no pain, and I don't feel bad. I have no reservations.” (26-year-old woman with a post-graduate diploma who has been married for four years)*

Another issue raised by several participants was anal sex.

*“Usually, after we flirt and get so excited because I feel pain with vaginal sex, we have anal sex.” “Interestingly, I do not have any pain with anal sex even in different positions, well, I don't have any problems, which is very surprising for me.” (37-year-old woman with a diploma who has been married for 6 years)*

### 3.2. Emotion-Focused Coping

All kinds of emotion-focused coping prevent a constructive or positive approach to the problem. It usually creates a negative emotional reaction that diverts a person's attention and ultimately does not lead to solving the problem. This category was created from 3 subcategories: a couple's negative reaction to the problem, attachment disorder, and surrendering the problem.

#### 3.2.1. Couple's Negative Reaction to the Problem

Many participants mentioned how couples reacted negatively to the problem. This category was created by combining the subcategories of penetration resistance, wife rejection when attempting vaginal penetration, and aggressive behavior of the husband during failed penetration.

Almost everyone interviewed expressed opposition to penetration.

*“When he wanted to be penetrated, due to the pain, I would put my hand on my husband's chest so that everything was under my control and I would not allow him to penetrate.” (25-year-old woman with a bachelor's degree who has been married for three years)*

Two of the participants in the current study mentioned their spouse's rejection when attempting vaginal penetration.

*“When we wanted to start, we were flirting and wanted to get into action, I would lock and he would get nervous and want to push.” “No, I said, don't do it because it bothers me. I'd get nervous and push him, then push him back and throw him. All I could think about was saving myself as soon as possible.” (38-year-old woman, elementary school, 10 years married)*

Another important issue raised by some participants is the husband's aggressive behavior during failed penetration.

*“My husband would get nervous just when I wasn't about to let him do penetration, he had finally reached the peak of pleasure, he wanted to be satisfied, he was waiting for me to be satisfied, then he could do it, it made him very nervous.” “Because his penis couldn't reach me when he wanted to go inside because I was pushing him back, he would get upset, yell, get up, and leave. He was cursing himself and blaming himself for initiating the intimacy, and he vowed never to do it again.” (41-year-old woman, bachelor's degree)*

#### 3.2.2. Attachment Disorder

Several participants stated that to deal with their sexual disorder, they keep their problem hidden from others and do not discuss it with others because they believe others do not understand their sexual disorder.

Several study participants mentioned hiding the problem.

*“Behind all my laughter was a pain that no one knew about except me, my God, and my husband.” (32-year-old woman, bachelor's degree, 4 years married)*

Several participants in the current study noted others' lack of understanding of their sexual problems.

*“I think that if someone looks at us from the outside, they might think of us as an abnormal person and they cannot understand this issue at all.” (28-year-old woman, bachelor's degree, 8-year marriage)*

Another issue raised by some participants was a failure to report the problem to others.

*“I never talked to anyone about this issue, to anyone, unfortunately, because of our society's culture; because I thought telling others would make me feel worse and would do nothing to help me.” (34-year-old woman with a bachelor's degree who has been married for two years)*

#### 3.2.3. Surrendering the Problem

Some participants admitted to giving in to their sexual problems. These people, according to themselves, constantly complained about their fate as a result of having this sexual problem, accepted their sexual problem as a permanent problem, justified their problem in various ways, and believed that their sexual problem was unique to them.

Several participants mentioned the term “fate.”

*“I keep telling myself, well, this is a disease, people don't have a special role in their disease, and you shouldn't blame yourself,” she says, “but I keep blaming myself, and I'm afraid of my fate and future.” (38-year-old woman, elementary school, 10 years married)*

Several participants mentioned accepting the disorder as a lifelong issue.

*“The same false information will set back a person's life by 5 years, 5 years will delay a problem and make you believe it will never be solved.” (30-year-old woman with a master's degree who has been married for 5 years)*

Another point raised by several people was the problem's justification.

*“I have a very good pain threshold, which is why I don't know why this problem happened to me.” (36-year-old woman with a Ph.D. who has been married for 5 years)*

The majority of participants emphasized the problem's uniqueness.

*“Hey, I was saying, God, there is no one like me, which means I am the only one who just turned out like this.” (49-year-old woman with a diploma who has been married for 18 years)*

### 3.3. Treatment-Seeking

This theme was created from 3 categories of searching and choosing a therapist to solve the problem, ineffective medical approaches, and ineffective nonmedical approaches.

#### 3.3.1. Searching and Selecting a Therapist

Some participants mentioned looking for and selecting a therapist to help them solve the problem. Participants in this study stated that they were constantly looking for a sex clinic and selecting the right therapist to solve their sexual problems, with the majority of them referring to gynecologists, sex therapists, psychotherapists, and midwives to solve their problems.

The majority of interviewees mentioned the sex clinic and finding the right therapist.

*“I searched a lot to find a female sex counselor, maybe that's why it took me so long to find one.” (38-year-old woman, bachelor's degree, 8-year marriage)*

The majority of participants in this subcategory sought treatment from a gynecologist.

*“I went to the gynecologist first to solve my problem because I didn't know who else to see.” (37-year-old woman with a diploma who has been married for 6 years)*

Some participants referred to sex therapist to solve the problem.

*“I went to a sex therapist and he told me that you have depression,” said one participant, “and because I was very upset by his words, I stopped having a good relationship with the sex therapist.” (35-year-old woman with a Ph.D. who has been married for three years)*

Some people in this subcategory used psychotherapy to solve their problems.

*“We went to a psychologist, and he told us to write down things like when you first discovered sex, where you had your first sex, what happened to you, or what you heard or saw about sex issues… Later, I attempted to write this. He teased me for a year about doing sexy movements in front of the mirror, but I realized it was pointless. My husband was also dissatisfied, so we decided to leave.” (31-year-old woman, master's degree, 3 years married)*

Going to a midwife to solve the problem was another option mentioned by several participants.

*“At first, my husband and I worked hard to get penetration, but it didn't work, so I went to the midwife, who checked and said there was no problem.” “And she assured me that there is no pain or fear.” (28-year-old woman, bachelor's degree, 8-year marriage)*

#### 3.3.2. Ineffective Medical Approaches

Several participants mentioned ineffective medical treatments. According to them, to solve their sexual problems, they turned to medical solutions such as Botox, exercise with unreasonable dilators, prescribing analgesia during penetration, taking sedatives during penetration, virginity surgery, RF and laser, biofeedback, and hypnosis, but none of these methods were effective.

Several current research participants stated in interviews that Botox is an inappropriate treatment option.

*“I did a lot of research on the Internet, and it was written that one of the ways is to do Botox.” When I was reading this, “I was telling myself that this was my only option, that at the very least the pain would go away and the penetration would be done properly, so that I would understand that nothing had happened at all. That is why I believe Botox could be beneficial.” (34-year-old woman with a bachelor's degree who has been married for two years)*

Some participants mentioned that exercising with unreasonable dilators is an inappropriate treatment strategy.

*“The first time I went to the gynecologist, she told me to do this with a carrot and put on a condom to make the hole in your vagina open, which I couldn't bear to do at all,” said one participant. (38-year-old woman, elementary school, 10 years married)*

Another treatment method mentioned by some participants was ineffectively administering analgesia at the time of penetration.

*“The majority of the doctors I saw told me to apply two Lidocaines, close my eyes, and do it.” “I bought and tried it. My vagina got numbed so I couldn't feel the penetration of my nail into the vaginal, however, I couldn't have sex penetration yet. I still had that guard from the inside all again.” (39-year-old woman, post-graduate diploma, 4 years married)*

Another issue that the majority of participants identified as an inappropriate treatment solution was the use of sedatives during penetration.

*“They gave me a lot of sedatives, one gave me diazepam, one gave me sleeping pills…, in short, everyone said something, but none of them worked for me.” “It only made me more afraid.” (38-year-old woman, elementary school, 10 years married)*

Another inappropriate treatment option mentioned by several participants was virginity surgery.

*“My husband believed that if I had surgery, my fear and pain would vanish and the problem would be solved.” In short, “I operated under anesthesia on my virginity and everyone thought I was fine… After a month of waiting for my wound to heal, we repeated the story.” (31-year-old woman, master's degree, 3 years married)*

Another inappropriate treatment option mentioned by several participants was RF and laser.

*“A series of experts told me to use RF laser.” I tried it, but it had no effect. I mean, “I went to 3-4 sessions, each of which cost me a lot of money, but it didn't help.” (29-year-old woman with a post-graduate diploma who has been married for four years)*

Another ineffective treatment strategy mentioned by several participants was biofeedback.

*“I went to 8 sessions of biofeedback along with pelvic physiotherapy and spent nearly 20 million, which was of no use to me, and I only became more and more depressed day by day.” (36-year-old woman with a Ph.D. who has been married for 5 years)*

Some participants also mentioned hypnosis as an inappropriate treatment solution.

*“We went to a doctor's office where she claimed that I will get better with a hypnosis session, but we were so stressed that it was of no use to us and we paid a lot of money, but in my opinion, she was a scammer. All she did was just talk.” (41-year-old woman, bachelor's degree, married for 6 years)*

#### 3.3.3. Ineffective Nonmedical Approaches

The experiences of participants in this category included three subcategories: drinking alcoholic beverages, seeking refuge in superstitions, and suggesting watching porn to solve the problem.

Another inappropriate nonmedical solution was the consumption of alcoholic beverages.

*“We were forced to try wine, which we did once, but it had no effect because the intensity of the pain and my fear were both so high that it didn't work.” (34-year-old woman with a bachelor's degree who has been married for two years)*

Several participants mentioned turning to superstitions for comfort.

*“I tried everything but it didn't work.” Finally, at the urging of my mother-in-law, I sought the services of a prayer writer. He wrote me a prayer and told me to act on Thursday by first putting this prayer in water and then drinking that water for both of you to establish a relationship, but it didn't work. (37-year-old woman with a diploma who has been married for 6 years)*

Another inappropriate nonmedical solution suggested by some participants was watching porn to solve the problem.

*“Several psychiatrists and counselors advised me to sit down with your husband and watch porn.” I even tried to take it, but I couldn't. “Maybe the porn movie will take away my fear, but will it take away my pain?!!!” (41-year-old woman, housewife who has been married for 6 years)*

## 4. Discussion

The current qualitative study sought to investigate the strategies for coping with sexual pain in Iranian women with GPPPD. Three main themes were found using content analysis: “problem-focused coping,” “emotion-focused coping,” and “treatment-seeking.”

The problem-focused coping consisted of the three categories of received social support, self-control, and penetration replacement. Some participants in the subcategory of received social support mentioned the spouse's emotional support, observance of the spouse in the sexual relationship, and the other's support. Consistent with current research, Bancroft has shown that one of the factors that cause two women with the same sexual problem to have different responses and sexual distress related to that problem is their emotional relationship with their spouse. A strong support network boosts self-confidence, self-esteem, and increased self-efficacy, which is linked to higher sexual satisfaction [23]. Couples who have more social support value sexual activity more are more likely to discuss their sexual problems and have higher sexual satisfaction [24]. The husband's and other family members' support influenced many affected women's perceptions of the need or lack of need for medical action, as well as their function. These supports, which included an insistence on action, verbal encouragement, financial assistance, and the provision of necessary facilities, all contributed to the treatment's success [25]. Therefore, it is clear that by receiving social support from others, especially the spouse, the amount of women's coping with their sexual problems increases.

Participants in this study reported taking actions such as muscle relaxation before penetration, avoiding negative thoughts, and attempting to increase sexual awareness to solve and escape from the problem in the subcategory of self-control. In fact, self-control is a psychological skill that allows a person to better control his or her emotions related to a problem and to better understand his or her inner abilities, which ultimately leads to individual peace [3, 26]. According to Reissing, 63% of women with primary vaginismus performed Kegel exercises to control their condition [27]. Kegel exercises, which aim to strengthen the pelvic floor muscles, aid in orgasm by increasing blood supply to the genital area and thus increasing arousal, as well as helping a person become more aware of and in control of the genital organ [28]. In one study, 52% of women with sexual pain disorders practiced relaxation exercises, while 32% sought sexual education to solve their problems [27]. It was demonstrated in the studies of Arslan-Özkan et al. that self-control reduces anxiety, depression, and stress and leads to better treatment outcomes. Women who do not know enough about themselves and their abilities, on the other hand, are at risk, which can lead to social isolation and harm their lives [29, 30].

Some participants in the subcategory of substituting penetration stated that they stopped sexual intercourse with foreplay, intercrural sex, oral sex, and anal sex to replace penetration. According to one study, women experiencing sexual pain learn to avoid many sexually arousing stimuli as a result of their pain and suffering, and if they are unable to avoid them due to the presence of a negative mentality caused by predicting the painful outcome again, it is unlikely to arouse the person. They avoid sexual intercourse, and if they are forced to do so, the person feels abused. Intimacy and closeness between the couple are replaced by discomfort, resentment, anger, and sadness [31]. On the other hand, a prohibition on sexual intercourse may result in the failure of the vicious cycle of sexual intercourse fear, followed by a negative experience, and finally, a sense of hopelessness in the patient, which leads to the cessation of sexual intercourse [32]. Given that some women with GPPPD disorders may attempt anal sex or other types of sexual activities to satisfy their sexual needs due to their inability to have vaginal sex. The current study's findings are consistent with those of Costa and Brody, who discovered that the avoidant attachment style of women who use dildos to achieve orgasm or who want anal sex is significantly higher [33].

The three categories of emotion-focused coping were the couple's negative reaction to the problem, attachment disorder, and surrendering to the problem. Some participants in the subcategory of couples' negative reactions to the problem mentioned penetration resistance, rejection of the spouse when attempting vaginal penetration, and aggressive behavior of the spouse during failed penetration. Women suffering from sexual pain disorders typically experience intense anxiety and stress with any penetration, resulting in extreme avoidance behaviors such as pushing or clenching their legs together and screaming [34]. Following these intense avoidance reactions, it is possible that the husband will misbehave. This misbehavior, which can include forced sexual intercourse, failing to pay attention to the wife's preparation, causing pain and pressure and humiliation, causes severe psychological pressure on women that is difficult to forget. Finally, if this psychological pressure is not alleviated over time, it will result in boredom and an aversion to sex [35]. In fact, the expression of anger, sadness, and anxiety during marital interactions may reduce marital satisfaction and compatibility in different ways [36].

Several participants in the attachment disorders subcategory mentioned hiding the problem, not understanding others about the sexual problem, and not reporting the problem to others. Given the negative social consequences of disclosing a problem, such as blame, social rejection, stigma, and even divorce for failed couples, women frequently choose to remain silent (in front of family, friends, and even therapists) about their sexual problems [37]. Some participants in a study conducted to explain the facilitators and barriers to seeking treatment in women with pelvic organ prolapse mentioned the shame of discussing reproductive and sexual problems. Because genital organs are very sensitive topics that people do not want to discuss, many affected women admitted that they had only spoken to a few close people about their problem [38].

Some participants mentioned complaining about fate, accepting the disorder as a permanent problem, justifying the problem, and the problem's uniqueness in the subcategory of surrendering to the problem. According to the evidence, many women with vaginismus express a conscious desire to perform penetration but involuntarily avoid it [39]. In various studies, most participants with sexual disorders stated that they believed they were the only ones with this problem until it was treated, and as a result, they avoided going to the doctor to solve their problem [40]; therefore, for these reasons, they surrendered to their sexual problem and did not seek treatment for their problem.

Treatment-seeking was divided into three subcategories: looking for and selecting a therapist to solve the problem, ineffective medical approaches, and ineffective nonmedical approaches. In this study, some of the participants mentioned researching the therapist and the sexual clinic and stated that they sought treatment from gynecologists, sex therapists, psychotherapists, and midwives. Women are more likely than men to seek treatment, medical consultation, and screening for health problems and diseases, according to studies, but in the field of diseases affecting the urinary-genital and sexual systems, women exhibit less treatment-seeking and self-care behaviors [41]. In a study of women with vaginismus, Reissing found that 76% were referred to a gynecologist or midwife, 65% to family medicine, 50% to a psychologist, 39% to a sex therapist, and 24% to a physical therapist. 13% had been referred to traditional medicine specialists, while 4% had been referred to other therapists [27]. In line with the current study, Reisy et al. stated that one of the main concerns and complaints of women with vaginismus was that they referred to gynecologists, midwives, and psychologists at different times to solve their problems, but in most cases, vaginismus was not diagnosed [42].

Most participants stated that medical approaches such as Botox, exercise with unreasonable dilators, prescribing anesthesia and anesthesia during penetration, taking sedatives during penetration, hymenectomy, radio frequency (RF) and laser, biofeedback, and hypnosis were ineffective and inappropriate in the subcategory of ineffective medical approaches. In fact, in various studies, hymenectomy has been mentioned as one of the unsuccessful treatment strategies [43]. The hymen is a mucous fold, not a curtain or a wall. Except in rare cases of imperforated hymen, hymen does not usually obstruct penetration. In most cases, genital penetration disorders are not anatomical disorders, and surgery that ignores the mechanism of these disorders not only fails to solve the patient's problem but also leaves her traumatized with visible physical changes. This problem can harm a person's mental health and leave them feeling inadequate [43]. During the years of suffering from these problems, couples were treated with a variety of ineffective treatments, including antidepressants, various benzodiazepines, water therapy, pain relievers, lubricant gels, numerous suggestions for performing sexual intercourse under anesthesia, and finally surgery. In addition to wasting time and money, these treatments had put a lot of psychological pressure on the patient, so that at the time of treatment, the patient was completely hopeless and suffered from depression, guilt towards his wife, suicidal thoughts, and the couple was in the divorce stage [44]. Therefore, the available evidence was in line with the findings of the present study regarding the ineffectiveness of these treatment strategies.

Several participants mentioned a subcategory of ineffective nonmedical approaches. They claimed that nonmedical approaches such as drinking alcohol, relying on superstitions, and watching porn were ineffective in dealing with the problem. According to the evidence, many of these couples resort to fortune-telling and exorcism to solve their problems, incurring significant financial costs in some cases [45]. According to Bokaei and Bostani Khalesi, some couples had the experience watching a porn movie before attempting penetration, which they described as a very unpleasant experience [44]. In addition, the ineffectiveness of consuming alcoholic beverages for the treatment of this disorder has been mentioned in various studies [37]. Based on the available evidence, all of the nonmedical approaches mentioned by the participants of this research are ineffective.

### 4.1. Limitations and Strengths

The narratives of women diagnosed with GPPPD were the focus of this study. Nonetheless, there are some limitations to the study. First, the qualitative nature of the methodology, as well as the resulting small sample size, limits the extent to which these results can be generalized. Of course, the sample data were saturated without any new issues emerging, which support the validity of our findings, which could have implications for many other sexual dysfunctions that affect women in settings similar to ours. Our study focused solely on the perspectives of women with GPPPD, with no consideration of the perspectives of male sexual partners. It is impossible to know what bias was introduced by the self-selection of participants. It is possible that the women who volunteered for the study were less knowledgeable about GPPPD and sought less assistance than the women who did not volunteer. However, qualitative studies do not seek to directly represent the specifics of large populations but rather to depict in some detail the lived experience of a few individuals in the hope that this experience is not out of the ordinary. The extent to which our findings would generalize to other cultures or even different national regions would have to be empirically tested.

Despite these limitations, the finding of this study suggests a sequence of experiences shared by the majority of women with GPPPD, at least generally. Furthermore, all members of the research team agreed on the interview script. Finally, this research was conducted in a specific cultural setting. It is an important path to understand because it indicates where we as healthcare professionals can to reach women who have significant sexual health problem. GPPPD public health information and education are required to catch these women before painful intercourse becomes an intractable presence for both patients and providers. Even if the consultation is for routine care, healthcare providers must step up and address the issue with sexually active patients. The investigation must then be supported by a delineation and discussion of multidisciplinary treatment options that foster proactivity, self-efficacy, and hope for problem resolution.

## 5. Conclusion

Coping strategies in women with GPPPD were classified as “problem-focused coping,” “emotion-focused coping,” and “treatment-seeking.” These findings indicate a need for GPPPD information and education, as well as a need for healthcare professionals to actively inquire about sexual problems and commit to serious treatment efforts. Cultural interventions that promote sexual pleasure can aid in the management of GPPPD. Therefore, there is a need to provide sexual health, counseling, and treatment services throughout the country's healthcare system by establishing, equipping, and expanding specialized centers with experienced staff.

## Figures and Tables

**Table 1:** Interview outline.

Items	
Before the interview	(1) Greeting, thanks, and introduction of the interviewer and the observer
	(2) Explained to the participants the purpose of the study
	(3) Explained the confidentiality of the information
	(4) Explained voluntary participation
	(5) Obtained written and verbal informed consent for study participation and voice recording

Questions for patients	(1) “What is your sexual disorder?” “Explain.”
	(2) What did your partner do to solve this problem?
	(3) What strategies have helped you to adapt to this disorder?
	(4) What strategies do you suggest to improve this disorder?

## Data Availability

The datasets of the present study are available from the corresponding author upon reasonable request.
